# Cutaneous Rosai-Dorfman disease with spontaneous partial involution

**DOI:** 10.1016/j.jdcr.2024.11.030

**Published:** 2024-11-30

**Authors:** Justin Porter, Sweta Subhadarshani

**Affiliations:** a University of Illinois College of Medicine, Peoria, Illinois; b Department of Dermatology, University of Pennsylvania, Perelman School of Medicine, Philadelphia, Pennsylvania

**Keywords:** acitretin, cutaneous Rosai-Dorfman disease, ILK, intralesional steroid, non–Langerhans cell histiocytosis, pruritus, retinoid, Rosai-Dorfman disease, sinus histiocytosis with massive lymphadenopathy

## Introduction

Rosai-Dorfman disease (RDD), also known as sinus histiocytosis with massive lymphadenopathy, is a rare form of non–Langerhans cell histiocytosis. It is characterized by a prolonged course of painless, bilateral cervical lymphadenopathy that typically self-resolves and may recur.^1^, ^2^, ^3^ This condition affects an estimated 1 in 200,000 children born in the United States each year and primarily affects the lymph nodes; however, 43% of patients with RDD exhibit extranodal disease.^2^^,^^4^ The most common sites of extranodal involvement include the skin, oral cavity, eyes, larynx, lower respiratory tract, bone, and central nervous system.^2^ Although the disease is generally benign and self-limited, some patients may experience complications related to organ involvement or mass effect.^4^ Isolated skin involvement occurs in 3% of RDD cases, termed cutaneous RDD (CRDD).^2^^,^^3^ Among these cases, recurrence has been documented to occur 4% of the time.^5^ Cutaneous lesions are frequently nonspecific, manifesting as multiple macules, papules, or plaques, with coloration ranging from yellow-red to red-brown. In cases of CRDD, only a single lesion may be present.^6^ Lesions are asymptomatic in 92% of patients, with tenderness and pruritus being the most commonly reported symptoms, occurring in 3.6% and 3.2% of cases, respectively.^5^ We present a case of isolated CRDD.

## Case report

**Fig. 1:**
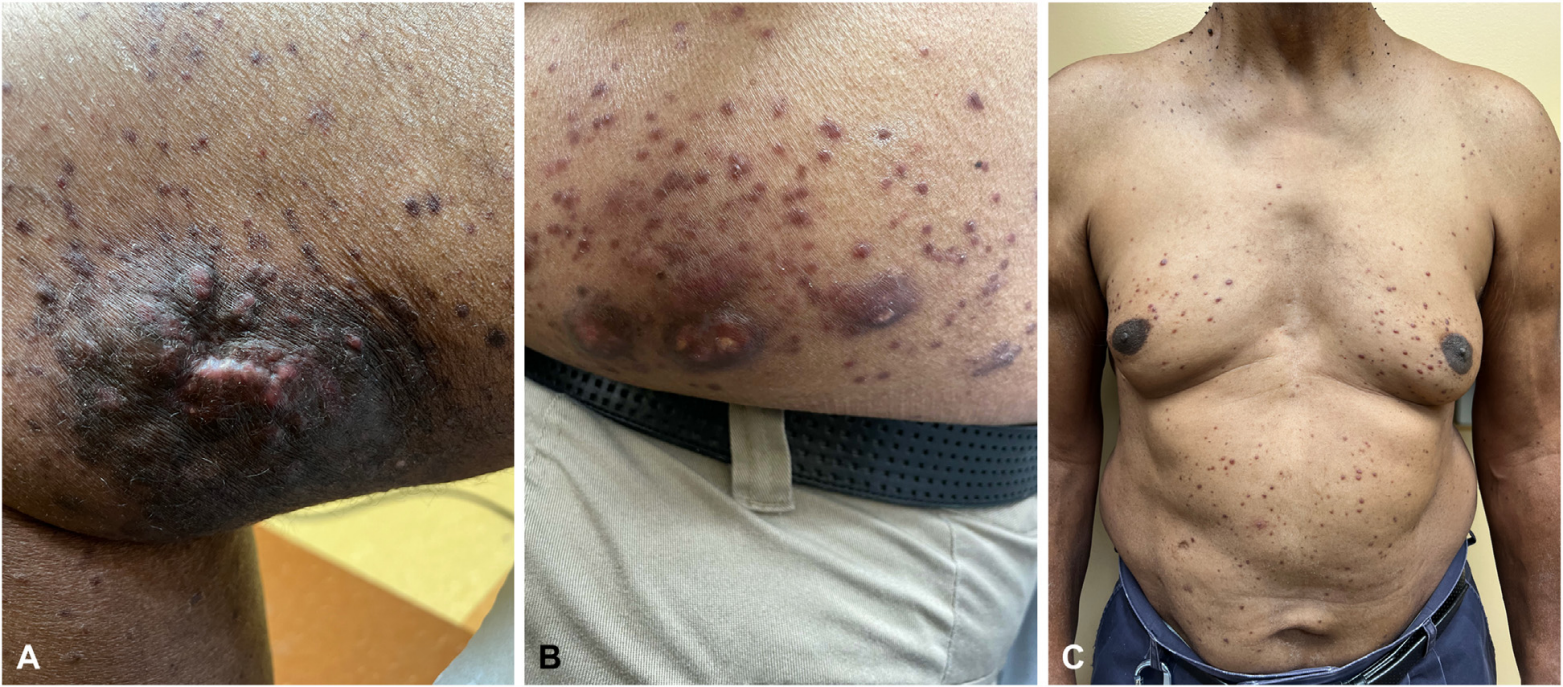
Pruritic hyperpigmented nodules and papules of cutaneous Rosai-Dorfman disease on the (**A**) right thigh and (**B**) left flank. **C,** Asymptomatic red-to-brown papules on the abdomen.

A 72-year-old man presented to the clinic with a 1-year history of a pruritic eruption on the right thigh and left flank (Fig 1, *A* and *B*) with recent progression over the previous 3 months with smaller lesions on the arms, legs, and trunk (Fig 1, *C*). He denied experiencing any constitutional symptoms, personal history of cancer, and recent medication changes.

Mucocutaneous examination was remarkable for multiple large, indurated, hyperpigmented to violaceous plaques involving the medial right thigh and left flank. Smaller, reddish-brown macules and papules were also observed on the trunk and extremities. No lymphadenopathy was appreciated upon examination.

Recent laboratory test results were notable for a hemoglobin concentration of 11.9 g/dL, a C-reactive protein concentration of 4.6 mg/L, and multiple M spikes on protein electrophoresis. Whole-body computed tomography was negative for lymphadenopathy but did incidentally reveal an asymptomatic solitary renal mass measuring <3.0 cm.

**Fig. 2:**
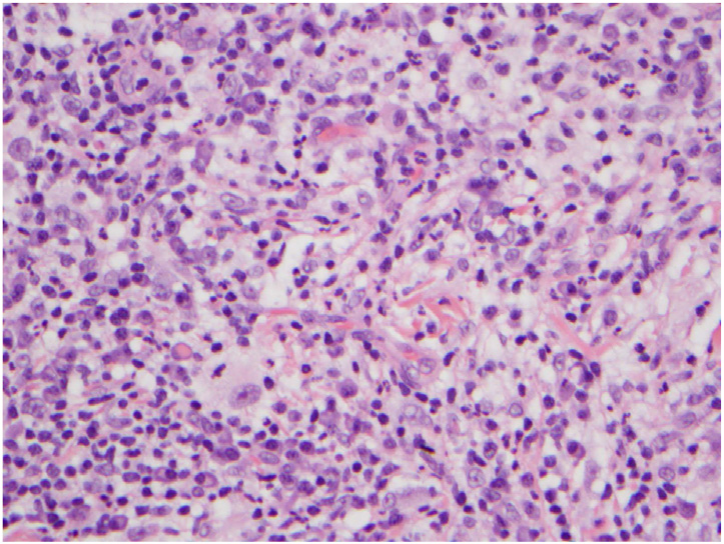
Large foamy histiocytes with prominent emperipolesis surrounded by a diffuse infiltrate of lymphocytes and plasma cells. (Hematoxylin-eosin stain; original magnification: ×40.)

Initial differential diagnosis included histiocytosis, sarcoidosis, and cutaneous lymphoma. Skin biopsy specimens were obtained, and histopathology revealed diffuse infiltration of soft tissue with a lymphoplasmacytic infiltrate showing germinal center formation. Clusters of histiocytes, characterized by eosinophilic/foamy appearance with emperipolesis (Fig 2), were further highlighted on immunohistochemical stain S100. Tissue staining was positive for S100, CD68, OCT2, and cyclin D1 but negative for CD1a. These findings were consistent with a diagnosis of extranodal RDD.

**Fig. 3:**
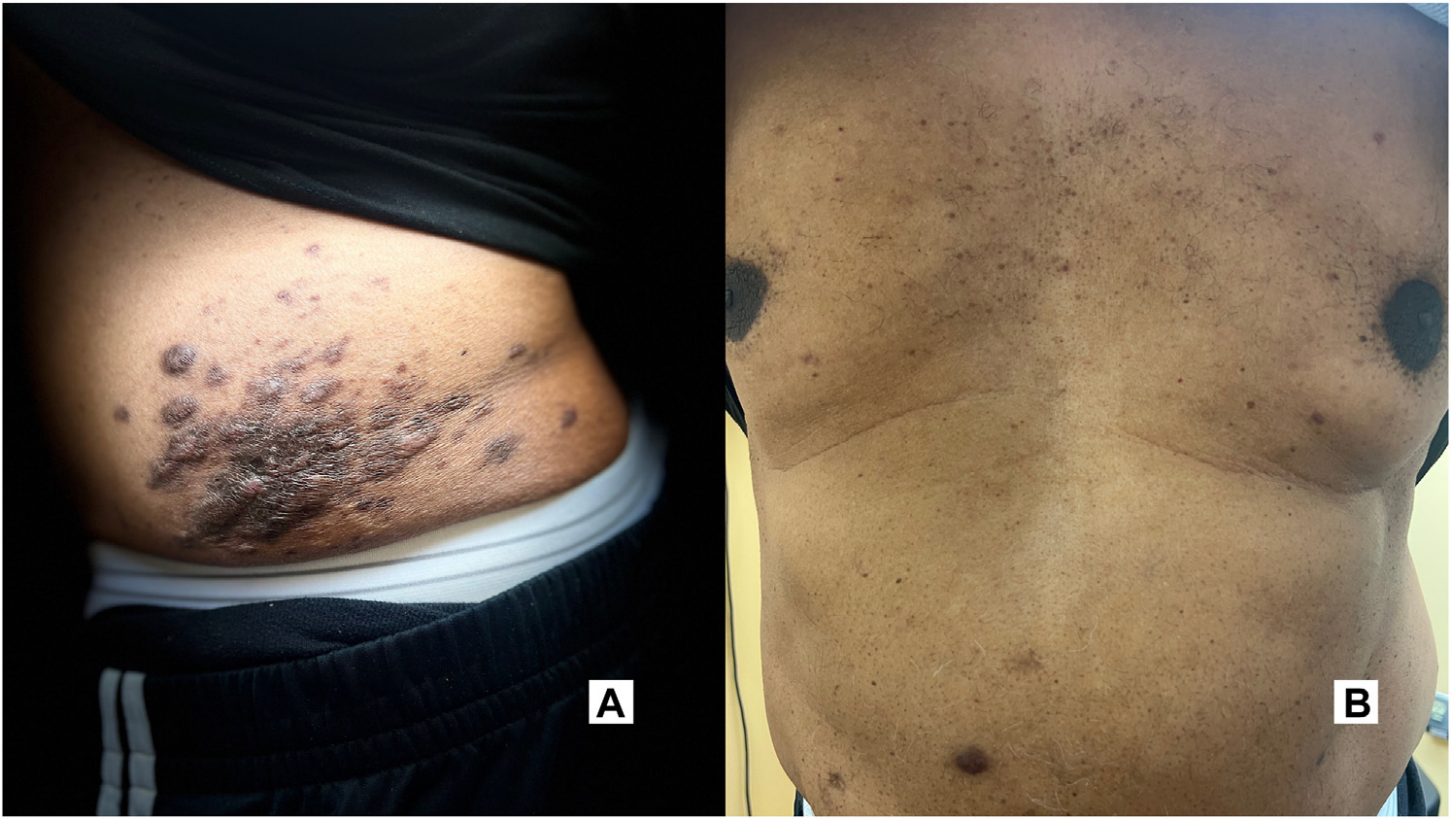
**A,** Persistent lesions on left flank and (**B**) clearance of lesions on abdomen at the 2-year follow-up.

The patient was treated with intralesional triamcinolone injection, which improved pruritus but not the appearance or consistency of larger lesions. He was also started on acitretin but could not tolerate it because of palmar peeling and severe dryness. The patient opted for observation after informed decision-making process. Gradually, the smaller lesions over his trunk completely resolved spontaneously, whereas the larger lesions remained persistent but asymptomatic at the 2-year follow-up (Fig 3, *A* and *B*).

## Discussion

The presentation of CRDD is often diagnostically challenging because lesions are frequently nonspecific, requiring histopathologic examination for diagnosis. Classically, histopathology demonstrates a dermal infiltrate of scattered large, pale histiocytes with prominent vesicular nuclei and abundant cytoplasm, termed “Rosai-Dorfman cells,” admixed in a perivascular infiltrate comprising lymphocytes and plasma cells.^7^ Emperipolesis, the histiocytic engulfment of inflammatory cells, of lymphocytes and plasma cells may be seen.^3^ The diagnosis is confirmed through immunohistochemical analysis of a skin biopsy specimen that reveals RDD histiocytes. These cells typically exhibit positive staining for S100, CD68, CD163, OCT2, and cyclin D1 markers while showing negative staining for CD1a, ALK, and langerin.^8^

The exact etiology of CRDD remains unknown, although it is hypothesized to involve an abnormal immune response or viral cause, such as Epstein-Barr virus, human herpesvirus 6, and herpes simplex virus.^5^ Pathogenesis is believed to involve macrophage colony–stimulating factor because it has been documented to induce macrophage lymphophagocytosis, an immunophenotype closely resembling the emperipolesis seen in CRDD.^9^

Other diagnoses to consider in the differential for CRDD include Langerhans cell histiocytosis, systemic RDD, malignant histiocytosis, cutaneous lymphomas, reticulohistiocytoma cutis, juvenile xanthogranuloma, metastatic melanoma, sarcoidosis, granuloma annulare, and a variety of infectious diseases.^1^, ^2^, ^3^, ^4^^,^^6^^,^^8^

To date, no proposed treatment plan has proven to be significantly efficacious. Current treatment options, with corresponding complete remission rates, include excision (59%), chemotherapy (22%), radiation (14%), oral or intralesional corticosteroids (5%), as well as cryotherapy and acitretin.^5^^,^^10^ Although most cases of CRDD are managed with 1 or more of the aforementioned treatment options, 24% of cases resolve without intervention.^5^ Given the benign nature and self-limited course of the condition, it is recommended to attempt less aggressive treatments first. Despite its rarity, awareness of RDD is essential for prompt diagnosis and appropriate management of affected individuals.

## Conflicts of interest

None disclosed.
